# Nutritional Characterisation of Childhood Chronic Kidney Disease: Trace Element Malnutrition in Paediatric Renal Disease (TeMPeReD) Study

**DOI:** 10.3390/nu17030535

**Published:** 2025-01-31

**Authors:** Matthew J. Harmer, Stephen A. Wootton, Rodney D. Gilbert, Caroline E. Anderson

**Affiliations:** 1 Southampton Children’s Hospital, Southampton SO16 6YD, UKcaroline.anderson@uhs.nhs.uk (C.E.A.); 2 School of Development and Health, Faculty of Medicine, University of Southampton, Southampton SO17 1BJ, UK

**Keywords:** CKD, paediatrics, nutritional assessment

## Abstract

Background/Objectives: In chronic kidney disease (CKD), poor nutrition is associated with poorer clinical outcomes. There are limited data on milder stages of childhood CKD. Methods: This study characterised the nutritional state of a cohort of children with CKD. Results: Within the cohort (mean age 10.5 years, mean eGFR = 57 mL/min/1.73 m^2^), obesity defined by body mass index rates was comparable to that in the general population, but central obesity (waist-to-height ratio > 0.5) was evident in 44% of children. Although average nutrient intakes for the cohort were acceptable, there was marked variability in the risk of poor nutrient intake (<LRNI): selenium (35%), magnesium (35%), iodine (30%), and zinc (30%). No child met the recommended dietary fibre intake. The prevalence of frank deficiency of vitamins and minerals in blood concentrations was low. Blood concentrations of vitamins A and E were near-universally elevated. In those who had a decline in kidney function at the 12-month follow-up, dietary intake of fibre correlated with the degree of decline. Conclusions: Much work is needed to optimise the nutritional status of children with CKD as an important modifiable risk factor for disease progression and other important outcomes.

## 1. Introduction

The incidence and prevalence of chronic kidney disease (CKD) in children are increasing, with a European incidence of stages 2–5 between 11 and 12 per million age-related population (pmarp) and a prevalence of 56–75 pmarp [1]. It is associated with an increased risk of altered dietary intake [2], nutritional inadequacy, suboptimal growth [3], and obesity [4,5]. Nutritional management is critical to multidisciplinary care, as poor nutritional status is associated with declining kidney function [6] and mortality [7,8].

There is little information on the nutritional status of paediatric CKD, particularly those with mild–moderate disease. Those publications that do exist are limited by a lack of complete nutritional characterisation, including dietary intake data [9]. Little is known about the demands for and losses of micronutrients in children across differing pathophysiological states or treatments of CKD [10]. It remains unclear whether an intake at or above the requirement of otherwise healthy individuals would be adequate in this patient group. The previous literature on biochemical measures of micronutrient status in paediatric CKD is limited to those with the most severe disease and those receiving dialysis, with contradictory findings [10,11,12,13].

In this Trace Element and Malnutrition in Paediatric Renal Disease (TEMPeReD) study, a cohort of children with predialysis CKD was nutritionally characterised, including the assessment of different measures of obesity and vitamin and mineral status.

## 2. Materials and Methods

The TEMPeReD study was a single-centre, cross-sectional study conducted to clinically and nutritionally characterise a cohort of children with CKD using anthropometric measures, dietary assessment, and blood concentrations of vitamins and minerals. Inclusion Criteria: aged between 3 and 17.99 years; CKD (stages 2–5); and informed consent. Exclusion Criteria: CKD stage 1; in receipt of dialysis; or renal transplantation.

### 2.1. Growth and Anthropometry

Height, weight, mid-upper arm circumference (MUAC), and waist circumference (WC) were obtained, with the subsequent calculation of body mass index (BMI), waist-to-height ratio (WHtR), height velocity, and, where appropriate, percentiles and standard deviation scores in comparison to reference standards [14]: Height SDS (HtSDS), Weight SDS (WtSDS), BMI SDS, MUAC SDS, and height velocity SDS (HtVelSDS).

### 2.2. Estimation of Dietary Intake

Dietary assessment was performed by a 24 h recall using a multiple-pass approach (an unstructured, uninterrupted listing of all foods and beverages consumed, followed by a structured approach to data collection including memory cues, and ending in an unstructured question for any other foods recalled, including several additional memory cues). Following the collection of dietary data, the foods and drinks were analysed using Netwisp computer software (Version 3.0, Tinuviel Software, Ltd., Bolton, UK), and nutrient composition was compared to dietary reference values for sex and age, including reference nutrient intake (RNI), lower reference nutrient intake (LRNI), or equivalents [15], and National Dietary and Nutrition Survey (NDNS) data as per their age grouping [16] that reflect the general population intake. The energy requirement was calculated using the Henry equation [17]. Recommended dietary fibre intakes were taken from the Scientific Advisory Committee on Nutrition (SACN) 2015 “Carbohydrates and Health” report [18].

### 2.3. Biological Samples

Blood and urine samples were collected from participants at the time of usual blood sampling. Analytes included serum creatinine to estimate the glomerular filtration rate (eGFR) and the blood concentrations of vitamins and minerals. Samples were analysed by the clinical science laboratory at a tertiary university hospital through their quality assurance framework and using their normal reference values. Blood measurements are reported as continuous variables and as “below reference range”, “within reference range”, or “above reference range”. The normal reference ranges are available in Table S1.

### 2.4. Follow-Up Data

Data (height, weight, and serum creatinine) were collected 12 months following baseline assessment through usual clinical follow-up.

### 2.5. Ethical Approvals

The study was approved by a Health Research Authority (REC:16/LO/0041). Informed consent was obtained from all individuals included in the study, with informed consent from caregivers and informed permission as appropriate.

### 2.6. Statistical Methods

Descriptive statistics, including the mean and standard deviation score (SDS) (or the median and interquartile range, IQR, depending upon the distribution of variables), were used to describe the data using appropriate tests for distribution (paired/unpaired *t*-tests, Mann–Whitney U (MWU), Kruskal–Wallis). Correlations with Pearson’s/Spearman’s rank tests were explored between growth measures, nutrient intakes, and nutrient blood concentrations and with other variables, including the level of deprivation (indicated by IDACI score [19]). All analyses were carried out using software from the Statistical Package for Social Sciences (SPSS version 22, SPSS Inc., Chicago, IL, USA). A *p*-value < 0.05 was used to indicate statistical significance. Bonferroni-corrected (α′= α/test number) [20] *p*-values are reported due to multiple comparators (p′).

## 3. Results

The cohort characteristics of the 46 participants are reported in Table 1.

### 3.1. Growth

**Figure 1:**
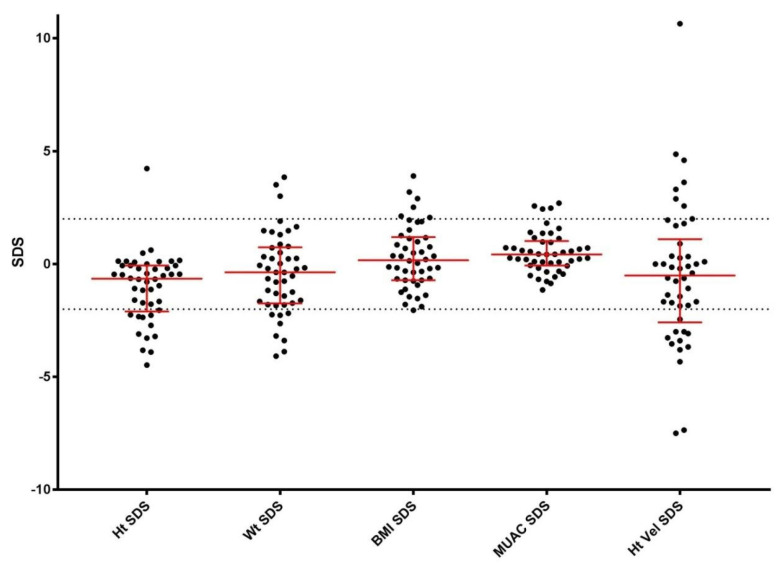
Baseline Anthropometry SDS. Standardised deviation scores for height, weight, body mass index, mid-upper arm circumference, and height velocity are shown for the entire cohort. Median scores with interquartile ranges are shown in red. The black dotted lines represent the normal reference range of −2 to +2 SD. Most children lay within the normal reference range for measurements, but compared to the reference standard, the group was shorter, with a large variation in height velocity. The variation in height velocity may represent the shifting of the normal growth curve to the right, with a delayed pubertal growth spurt and/or catch-up growth following. Abbreviations: BMI—body mass index; Ht—height; Ht Vel—height velocity; MUAC—mid-upper arm circumference; SDS—standardised deviation score; Wt—weight.

Growth measurements are shown in Figure 1. Eight children (17%) had WtSDS < −2 (malnourished by ICD-10 definition), and three children (7%) had WtSDS > 2. One child (2%) was underweight-for-height (BMI SDS < −2); thirteen children (28%) were at risk of being overweight (>85th percentile); and six children (13%) had BMI SDS > 2. Twelve children (26%) were short-for-age (HtSDS < −2), and one child (2%) was tall-for-age (HtSDS > 2). Twenty children (44%) had a WHtR > 0.5. The median HtVelSDS was −0.55 (IQR ± 2.5), with 28% having an HtVelSDS < −2. One child was receiving growth hormone treatment. HtSDS and BMI SDS demonstrated a positive correlation (*S.rho* = 0.568, *p* < 0.0005).

There was no difference between those with HtSDS < −2 and those with HtSDS > −2 in age, time since diagnosis, number of medications, eGFR, degree of proteinuria, dose of angiotensin-converting enzyme (ACE) inhibitor (mg/kg), C-reactive protein, or level of deprivation (IDACI score). There was no difference in HtSDS when comparing CKD stage 2 and other CKD stages.

Short-for-age children had lower median parathyroid hormone (PTH) concentrations (2.7 pmol/L (IQR ± 1.5) vs. 5.7 pmol/L (IQR ± 7.9), MWUT = 91.5, *p* = 0.038) and higher median serum albumin (42 g/L (IQR ± 3.75) vs. 39.5 g/L (IQR ± 4.5), MWU t = 262, *p* = 0.022).

If grouped as either (i) HtVel SDS < −2 or (ii) HtVel SDS > −2, there was a significant difference in eGFR, with those with HtVelSDS < −2 having higher mean eGFR (65.5 SD ± 24.6 mL/min/1.73 m^2^ vs. 49.8 SD ± 21.1 mL/min/1.73 m^2^, *t*-test t(44) = 2.329, *p* = 0.025). There was no difference between groups in the degree of proteinuria or medication burden. Those with HtVelSDS < −2 were younger (8.5 SD ± 3.6 vs. 12.4 SD ± 3.9 years; t(44) = −3.495, *p* = 0.001) and more recently diagnosed (73.7 SD ± 49.5 vs. 109.2 SD ± 55.7 months; t(44) = −2.277, *p* = 0.028).

#### Obesity

When comparing different definitions of obesity, the prevalence of obesity in the cohort was BMI SDS > 2 = 13%; WHtR > 0.5 = 41%; WC > 90th centile = 11%. Kappa-agreement values between the definitions were BMI SDS > 2/WHtR > 0.5 κ = 0.351; BMI SDS > 2/WC > 90th centile κ = 0.484; WHtR > 0.5/WC > 90th centile κ = 0.111. There was no difference in the rate of BMI-defined or WHtR-defined obesity between sexes, nor in eGFR. There was no difference in BMI SDS when comparing CKD stage 2 and other CKD stages.

### 3.2. Dietary Intake

Dietary intakes of energy, nutrients, and dietary fibre, compared with recommended intakes and NDNS data of intakes for the general population, are reported in Table 2. Mean energy intake, potassium, magnesium, iron, iodine, selenium, zinc, and vitamin K were below recommended minimum intakes (RNIs or equivalent). The nutrients that had the greatest number of intakes below LRNIs (most at risk for inadequacy) were selenium (35%), magnesium (35%), iodine (30%), zinc (30%), and potassium (24%). No participants met the recommended intake of dietary fibre.

Twenty-eight children (70%) received some form of nutritional supplementation, such as feed, sip-feed, or supplements (details of the dietary products for the cohort are given in Table S2). The most frequently prescribed nutritional support was iron as ferrous sulphate or ferrous fumarate (37%) and vitamin D supplements as cholecalciferol (28%). Two families had chosen to administer their own choice of “over-the-counter” vitamin preparations (one a multivitamin, and one a vitamin C supplement). Those in receipt of sip-feed supplementation were more likely to be short (HtSDS < −2) (***χ***-squared = 8.803; *p* = 0.003).

The mean sodium intake was no different from the general population intake and greater than the recommended maximum intake for those aged 4 to 10 years. Despite the average intake of older children being lower than the recommended maximum, nearly half of the children had intakes greater than this maximum, with a greater number of younger children exceeding it compared to older children. Most children were taking anti-hypertensive agents (all enalapril), with four children taking multiple agents (other agents were amlodipine (four) and atenolol (one)). There was no difference in sodium intake in those receiving anti-hypertensives and those not (180 SD ± 113%RNI vs. 158 SD ± 83%RNI, respectively). The dose of enalapril (mg/kg) did not correlate with sodium intake. The number of children exceeding the maximum sodium intake was similar between those taking anti-hypertensives and those not. Dietary intake of sodium did not correlate with blood pressure SDS for those not prescribed anti-hypertensive agents.

There was no difference in sodium intake depending upon diagnosis (Kruskal–Wallis test t(12) = 14.915, *p* = 0.246), with no difference between dysplasia patients and all other diagnoses (MWU t = 129.00, *p* = 0.105). The number of children exceeding the maximum sodium intake was similar between those with a diagnosis other than kidney dysplasia (that may be associated with renal sodium wasting) and the group as a whole.

Compared to the general population intake (NDNS data presented in two age groups; see Table 2 and Tables S2 and S3), the younger CKD subgroup had lower potassium and greater vitamin A and zinc intakes. In the older CKD subgroup, the cohort had lower energy, protein, potassium, magnesium, iodine, selenium, and zinc intakes compared to the NDNS data. If Bonferroni correction was applied, potassium (p′ = 0.012), magnesium (p′ = 0.013), selenium p′ = 0.007), and zinc (p′ = 0.019) remained significantly lower, but only in the older age group.

Age was greater in those with intakes < RNI (zinc: MWU t = 356, *p* = 0.026; iodine: MWU t = 381.5, *p* = 0.007; iron: MWU t = 370.5, *p* = 0.02). Given this, correlations were sought between age and dietary intakes of nutrients. The strongest negative correlations were between age and manganese (*S.rho* = −0.632); selenium (*S.rho* = −0.618); vitamin B12 (*S.rho* = −0.515); vitamin K (*S.rho* = −0.479); and folate (*S.rho* = −0.413). Less dietary fibre as a percentage of recommended intake was consumed with increasing age (see Table S4). Although iron and copper intakes were lower in those with lower dietary fibre (see Table S5) and their intakes correlated with one another (*S.rho* = 0.700, *p* < 0.001), there was no correlation between dietary fibre intake and iron (*S.rho* = −0.057, *p* = 0.740) or copper (*S.rho* = −0.189, *p* = 0.269) intake.

### 3.3. Blood Vitamin and Mineral Concentrations

The blood concentrations of vitamins and minerals are reported in Table 3. All children had at least one biochemical measure of micronutrient status that fell outside of the normal reference range, and the median number of abnormal blood results per child was 2.5 (IQR ± 1). The most common abnormal measurement was an elevated vitamin E concentration (95%), followed by elevated vitamin A (74%), elevated manganese (23%), low zinc (12%), and low vitamin D (13%). There was a low prevalence of blood concentrations of micronutrients below the normal reference ranges: vitamin A (2%), vitamin D (13%), manganese (6%), selenium (2%), and zinc (12%). Other micronutrient concentrations below the lower limits of the reference ranges were vitamin B6 (2%), vitamin B12 (2%), vitamin C (2%), and copper (2%). No patients had vitamin E or folate measures below the lower limit of the reference range. There was no discernible pattern of abnormal micronutrient concentrations grouping individuals and no differences between sexes.

A similar pattern of blood measurement abnormalities was seen in those with mild (CKD stage 2) compared to those with moderate–severe disease (CKD stages 3 and 4). However, elevated vitamin A was more prevalent in those with more severe disease, and eGFR was found to inversely correlate with vitamin A concentration (P.coeff = −0.649; *p* < 0.0005). In contrast, biochemical measures of vitamin B12, selenium, and manganese concentrations showed positive correlations with eGFR: vitamin B12 (*S.rho* = 0.321, *p* = 0.044), selenium (P.coeff = 0.309; *p* = 0.047), and manganese (*S.rho* = 0.353, *p* = 0.037).

#### 3.3.1. Vitamin A

**Figure 2:**
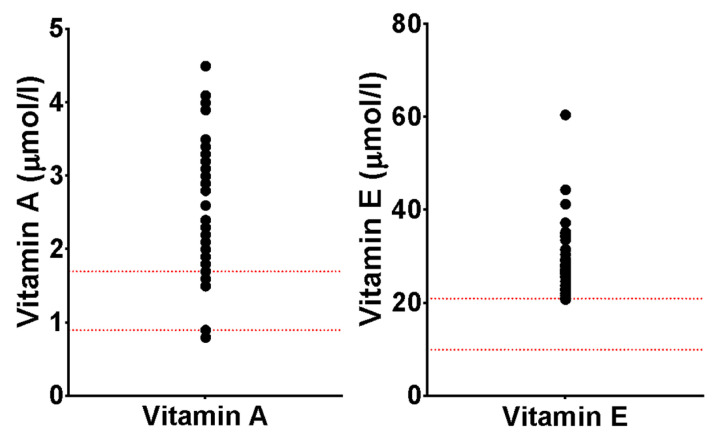
Distributions of blood concentrations of vitamins A and E. Reports the distribution of vitamin A (total retinol) and vitamin E (total tocopherols) in the cohort, with the normal reference ranges indicated by the dotted lines. Both vitamins were elevated in the majority of children, with a range of values up to 3 times the upper normal reference range. The clinical significance of this has yet to be determined in children with chronic kidney disease.

Most children had elevated vitamin A concentrations (74%) (see Figure 2). Twelve (26%) children received nutritional support that contained vitamin A, 10 (22%) being in the form of a sip-feed/supplement rather than a feed. The prescription of nutritional support containing vitamin A or a sip-feed or supplement did not correspond to higher vitamin A concentrations (t(40) = −0.917, *p* = 0.365; t (36.146) = 1.014, *p* = 0.317; and t(28.781) = 0.578, *p* = 0.568, respectively). Vitamin A concentration did not correlate with albumin-corrected calcium concentration (P.coeff = −0.051, *p* = 0.747). Serum vitamin A concentration negatively correlated with Hb concentration (P.coeff = −0.499, *p* = 0.001), with higher vitamin A concentrations in those with Hb < 120 g/L (3.12 SD ± 0.80 vs. 2.18 SD ± 0.75, t(40) = −3.313, *p* = 0.002).

#### 3.3.2. Vitamin E

As shown in Table 2, most children had elevated vitamin E concentrations (95%). There was no difference between those who were deemed at risk of obesity (BMI SDS > 2, or WHtR > 0.5) and those who were not (BMI: MWUT = 27.00, *p* = 0.714; WHtR: MWUT = 122.50, *p* = 0.124). Of the 46 children, 25 children had (non-fasted) cholesterol profiles available (54%), with a mean total cholesterol of 4.56 mmol/L (SD ± 1.08). Twelve children (48%) had total cholesterol concentrations > 4.40 mmol/L (borderline), and six (24%) had high levels (>5.15 mmol/L). The mean non-HDL cholesterol concentration was 3.06 mmol/L (SD ± 1.14 mmol/L; borderline/high (>3.18 mmol/L) = 10 (40%); high (>3.73 mmol/L) = 4 (16%). Total or non-HDL cholesterol concentration did not correlate with vitamin E concentration (*S.rho* = 0.359, *p* = 0.092; and *S.rho* = 0.308, *p* = 0.152, respectively). A total of 48% had a vitamin-E-to-cholesterol ratio > 90th percentile.

#### 3.3.3. Folate

Those receiving folate supplements had significantly higher serum folate concentrations (median = 25.00 IQR ± 7.60 vs. 10.7 IQR ± 9.86; MWUT = 129.00, *p* = 0.004), and three out of five high values were in those taking supplements. No children were taking medication known to alter folate absorption or metabolism.

#### 3.3.4. Manganese

Whole-blood manganese concentrations were high in 23% of children and low in 6%. Those with Hb < 120 g/L had higher whole-blood manganese concentrations than those with higher Hb (median = 128.00 IQR ± 66.00 vs. 169.00 IQR ± 106.75; MWUT = 62.5, *p* = 0.038). There was no correlation with Hb or mean corpuscular volume (*S.rho* = 0.097, *p* = 0.578 and −0.305, *p* = 0.075, respectively) and no difference in manganese concentration between those in receipt of iron supplements and those not (MWUT = 143.5, *p* = 0.907).

### 3.4. Follow-Up Data at 12 Months

Data (height, weight, and serum creatinine) were collected at 12 months following baseline assessment through the usual clinical follow-up. A change in height SDS of 0.2 was a priori determined to likely represent an actual deviation in SDS. Of the original 46 participants, 42 had available 12-month measurements, and of these, “no change”, “a decline”, and “an increase” in height SDS were seen in 27 (64%), 5 (12%), and 10 (24%), respectively. Paired *t*-test analysis for HtSDS at baseline and 12 months showed a significant difference, with a mean decline in height SDS of 0.13 (SD ± 0.42, t(41) = −2.025, *p* = 0.049).

When examining the subgroup of those with poor growth at baseline (HtSDS < −2, n = 11), a greater proportion of children increased their HtSDS (no change = 2 (18%), decrease = 0 (0%), increase = 9 (82%)).

The median change in eGFR from baseline to 12 months was available for 36 participants and was 1.02 mL/min/1.73 m^2^ (IQR ± 12.6) (mean = 0.39, SD ± 9.12). There was no association between the change in kidney function and the change in HtSDS. In those who had a decline in GFR at 12 months (n = 19), lower dietary fibre intake was associated with a greater decline in eGFR (*S.rho* = 0.890, *p* < 0.001). When comparing those below and above the median dietary fibre intake, there were no significant differences in HtSDS, WtSDS, or BMI SDS at follow-up (see Table S5).

When comparing those with stable eGFR and those with a decline at 12 months, intakes of energy and vitamin C were lower in the stable group (energy % of requirements: 74% vs. 99%, MWUT = 231.5, *p* = 0.025; vitamin C % of requirements: 120% vs. 206%, MWUT = 224, *p* = 0.048), and vitamin B6 was higher (220% vs. 160%, MWUT = 96.0, *p* = 0.038) (see Table S6).

## 4. Discussion

Nutritional status may be compromised in children with CKD. In the TEMPeReD study, a cohort of children with predialysis CKD has been clinically and nutritionally characterised. It provides contemporary data that include dietary intake estimation, blood vitamin and mineral concentrations, and several measures of obesity. This analysis has demonstrated that about a quarter of the cohort comprised short-for-age children. Despite normal to elevated BMI and normal ranges of nutrients in the blood, this is not explained by disease severity alone. The prevalence of obesity, as defined by BMI, is similar to that in the general population (13%), but abdominal obesity (WHtR) was much greater than this (44%).

In agreement with the general childhood population, older children with CKD were at greater risk of inadequate intake. No child met the recommended dietary fibre. Moreover, dietary fibre intake was associated with a decline in eGFR at 12 months. Despite the high prevalence of children at risk of nutrient inadequacy, the blood concentrations of vitamins and minerals generally lay within the normal reference ranges, except for near-universally elevated serum blood concentrations of vitamins A and E.

### 4.1. Growth

Several measures can be used to characterise growth. In the TEMPeReD cohort, a significant proportion were short-for-age (HtSDS < −2 = 26%), and the same proportion had poor linear growth (HtVelSDS < −2 = 28%), and 10% of the cohort had poor growth by both definitions. In this cohort of predialysis paediatric patients, growth patterns can be divided into three broad populations: (1) those with height and BMI within the normal limits; (2) those who are short in stature but with a normal BMI; and (3) those with an elevated BMI, but with an average height. The largest proportion of patients fell into the “normal height and BMI” category, and there were no children that fell into the traditionally described anthropometric phenotype of the “short and skinny” CKD patient. Instead, those falling out of the “normal” parameters were short-for-age (HtSDS < −2) or heavy-for-height-and-age (BMI SDS > 2).

Higher serum albumin concentrations in those short-for-age may represent improved nutritional status due to the recognition of growth problems but without an improvement in HtSDS. PTH concentrations were lower in those who were short-for-age, although the two patients with PTH concentrations below the normal reference range were normally grown. Other markers of disease severity did not demonstrate differences. PTH concentrations may reflect bone turnover. However, as the values lay within the normal reference range, it is difficult to be convinced of the clinical significance of the observed differences. The effect of short-for-age is long-lived, with the first 1000 days of life having the greatest impact, with less capacity for catch-up growth after that. In this cohort, there may be limited ability to alter the trajectory of these short-for-age children, who will remain so into adulthood.

### 4.2. Obesity

Obesity rates in the TEMPeReD cohort compare similarly to the general UK population [21]. Obesity is becoming increasingly prevalent in CKD populations across the UK and Europe [5] and more prevalent than the canonical malnutrition state of low anthropometric scores and “protein-energy wasting” in both the predialysis [4] and dialysis/post-transplant populations [5]. It has been reported to be an independent risk factor for the development of kidney damage and end-stage kidney disease [22,23]. The reasons behind the obesity levels in this cohort are likely similar to those in the general population, with increased availability of nutrient-poor, energy-dense foods and the potential for different physical activity levels. In those with CKD, it may be that dietary patterns are similar to those in the general population, especially in the early (milder) stages of the disease, where there is less dietetic input, and physical activity levels may be lower than in healthy peers due to the effects of the disease.

Alternative markers of obesity and cardiovascular risk include markers of central adiposity/obesity (for example, WHtR > 0.5 and WC > 90th centile). More children (35%) had abdominal obesity (WHtR > 0.5) than BMI-defined obesity. This discrepancy may be because BMI is under-representing the degree of adiposity. In CKD, it is well recognised that there is a loss of body mass (“protein-energy-wasting”), which is thought to be secondary to chronic inflammation. There was a similar prevalence of obesity as defined by BMI and WC > 90th centile, but not in the same individuals. In-office measures that more accurately reflect adiposity and associated risk are needed, and WHtR is worth exploring in future body composition studies, for example.

### 4.3. Dietary Intake

There was large variation in the risk of nutrient intake, with a large proportion at risk of inadequate intake (<LRNI) of selenium (35%), magnesium (35%), iodine (30%), and zinc (30%). Despite this, only zinc and dietary fibre intakes were significantly below the recommended levels for average intakes for the cohort.

Compared to the general population, the cohort had a higher proportion of children with estimated intakes below the LRNIs for several nutrients. Even compared to the general population, in which many nutrients have a high prevalence of inadequate intakes, average TEMPeReD intakes in those aged 11 to 18 years were lower for magnesium, potassium, selenium, and zinc; this is despite the general population having mean intakes lower than the RNIs.

Lower energy and vitamin C and higher vitamin B6 intakes were associated with a lack of decline in kidney function at 12 months. This may be explained by dietary modification and dietitian input being more likely/intensive in those with more progressive disease, resulting in the promotion of energy and vitamin C intakes but the limitation of protein sources, which are also vitamin B6 sources.

In more economically developed countries’ diets (including the UK), salt intake is greater than is required and is above the recommended intake levels [18], above which poorer health outcomes (hypertension and associated cardiovascular disease, including death from a stroke) are associated [24,25]. Younger children tended to exceed the maximum dietary sodium intake, in contrast to older children. The blood pressure of the cohort was well controlled despite this. Despite regular medical review and guidance, a behaviour change to decrease sodium intake is needed. As much of the sodium intake of the UK population arises from processed foods, an increased awareness of this must be instilled in the families of our population to empower them to make healthy food choices. At the population level, efforts must be pursued to decrease the salt content of foods available through public health strategies.

Current guidance recommends between 15 g and 30 g per day of dietary fibre [18]. There is significant evidence for the benefit of a high-fibre diet in the general population, with much epidemiological data demonstrating a relatively reduced risk of cardiovascular disease in those with higher intakes of dietary fibre [26,27,28,29]. In the data presented here, no child or young person had a dietary intake of fibre that met these recommendations, with the average intake for the cohort being only ~35% of the recommended intake. There was a pattern indicating that younger children had intakes closer to their recommendations (although still less than half of the recommendation intakes). This is in keeping with adult CKD patients not meeting recommendations [30] and national UK data from the NDNS survey of the general paediatric population. In the NDNS dataset, fibre intakes were 14 g/d and 15.3 g/d for age groups 4–11 and 11–18 years, respectively [16], greater than the intakes reported in this study. So, the paediatric CKD population may be particularly vulnerable to not meeting their recommended fibre intake, possibly due to dietary modifications.

With these data in mind, the next question to be asked is whether dietary fibre supplementation is warranted in the paediatric CKD population. Dietary fibre has been demonstrated to alter transit times, alter satiety, act as an energy source for the gut microbiota, and affect the innate immune system of the gut mucosa both directly and indirectly [31]. Supplementation with fibre in CKD patients has been associated with increased colonic bacteria and a decrease in serum urea of 12% [31]. NHANES data from the USA have associated lower markers of inflammation (CRP) and mortality with higher fibre intakes in the adult CKD population [30]. In agreement with the previous literature, in children with a decrease in their kidney function at 12 months, those with lower estimated dietary fibre intakes had the greatest decline. More effort is needed to increase dietary fibre intake in children (including those with CKD).

Regarding blood concentrations of vitamins and minerals, this cohort of children had few overt biochemical signs of poor vitamin and mineral status. The exceptional measures were serum concentrations of vitamins A and E, which were consistently markedly elevated above the reference ranges, even in those with less severe disease, despite limited oral micronutrient supplementation. Vitamin A concentration had a strong negative correlation with kidney function. Joyce et al. [13] reported micronutrient data in their paediatric dialysis population, showing a high prevalence of elevated vitamin A and higher average concentrations than this cohort. Elevated concentrations are unlikely to be due to excessive intakes, increased absorption (already high in healthy children), or decreased losses (already low in healthy children). It is, therefore, likely due to altered metabolism (increased hepatic mobilisation due to increased retinol-binding protein that is usually metabolised in the kidney or decreased uptake from peripheral tissues). Chronic hypervitaminosis A can cause several signs and symptoms that are seen in the CKD population. These include anorexia, nausea, vomiting, and an increased risk of bone fracture. The aetiology of these symptoms in those with CKD is multifactorial, including uraemia and mineral-bone disease of CKD, but it may be that a degree of elevated vitamin A blood concentrations also contributes. Moreover, vitamin A regulates bone turnover, with excessive vitamin A resulting in decreasing bone mineral density through the inhibition of osteoblasts and the stimulation of osteoclasts. Elevated vitamin A is associated with hypercalcaemia [32] and has been observed in both adults receiving dialysis [33,34,35] and paediatric dialysis patients, where retinol-binding protein correlated with hypercalcaemia [36]. The data presented here did not show an association between vitamin A and albumin-corrected calcium concentrations, although there are many factors that may mask a potential association, and retinol-binding protein was not measured.

Although there is recognition that elevated vitamin A concentrations are common in CKD, there are limited therapeutic options. Current dietetic practice is to choose feeds and supplements that have the lowest vitamin A within them. The limitation of vitamin A intake (removal of vitamin A-containing supplements) in haemodialysis patients has been shown to decrease serum vitamin A concentrations [35]. There is currently no evidence that an elevated vitamin A concentration, except at the extreme end of toxicity, has an impact upon outcomes. Moreover, limiting vitamin A intake below requirements to decrease blood retinol concentrations may have negative consequences on health.

A high proportion of children had increased vitamin E concentrations (95.2%). Vitamin E has been previously reported as low [11,37], normal [12,38,39,40], and high [13]. The data reported in this study suggest that vitamin E is elevated in CKD, agreeing with Joyce et al.’s [13] reported micronutrient data in their paediatric dialysis population showing a high prevalence of elevated vitamin E in paediatric dialysis patients and higher average concentrations than in this cohort; this suggests the possibility of a “dose–response” relationship between disease severity and vitamin E, although, in these data, the correlation was not statistically significant. Similarly to the elevations in vitamin A, the increased concentrations observed are likely to be due to metabolic alteration, and as the main source of vitamin E is adipose tissue, it may be linked to the altered lipid-handling in CKD.

Vitamin E measurement is affected by fasting/non-fasting status and may explain some of the inconsistencies in the literature. Additionally, CKD is associated with dyslipidaemia, and although only a subset of the cohort had cholesterol profiles available, the prevalence of dyslipidaemia is similar to that reported in the CKiD cohort, in which a prevalence of 45% is reported [41]; this is not high enough to explain the high vitamin E status found, especially as this cohort had better eGFR (57 vs. 43 mL/min/1.73 m^2^). The findings were not associated with markers of obesity. Not all the vitamin E concentrations were adjusted for cholesterol status, but in about half the subgroup where this was possible, cholesterol-adjusted vitamin E concentrations were greater than the 90th percentile. Excess vitamin E is associated with coagulopathy and decreased platelet function [42], and trials of high-dose supplementation have demonstrated an increase in all-cause mortality [43,44]. Unfortunately, this study did not measure coagulation or leucocyte function in order to evaluate this possible negative impact. Future work should examine this. Vitamin E toxicity can also cause nausea, fatigue, and muscle weakness, symptoms that are also seen in those with CKD.

The limitations of this study include the observational nature of the TEMPeReD study and the lack of a control group; it could not establish that there was a cause-and-effect relationship between the variables. This study was limited by the number of subjects due to the rarity of CKD in childhood and the associated wide age range, as well as the lack of socioeconomic determinant data. All dietary recall methodologies are limited in their ability to reflect true intake. This is especially true for vitamins, minerals, and trace elements, which are infrequently taken. Although more arduous for participants, prospective food diaries and, when available, food frequency questionnaires may more likely reflect true intake. The application of dietary intake thresholds such as LRNI to the individual patient has limitations, as this represents a statistical likelihood for a requirement, rather than the patient’s personalised requirement. Blood concentrations of vitamins and minerals were single values, offering a snapshot for the patient, and do not give the details of changes over time, for example.

Future studies should focus on understanding the optimal measures of adiposity and the true dietary intake requirements for children with CKD rather than relying upon the recommended intake for healthy children (current practice). Exploring functional markers of vitamin and mineral status may facilitate this. As this study is limited by its size, national or international studies with longer follow-ups that would allow for greater exploration of age groups, for example, are needed. This may be facilitated through the expansion of national registry data, which are currently limited to the latter stages of CKD and do not examine nutritional status above growth data and do not look at individual patient growth trajectories. Additionally, exploring the inflammasome and oxidative stress interaction with nutritional status should be included. A specific study examining socioeconomic determinants of nutritional status, including food security, access to healthy foods, and dietary counselling, is also needed to explore health inequalities in this area.

## 5. Conclusions

In conclusion, the TEMPeReD study offers a multifaceted nutritional characterisation of children with CKD. Although limited by the rarity of childhood CKD, it highlights challenges with growth, obesity (particularly central obesity), and dietary fibre intake (which may have an impact on CKD disease progression). Blood concentrations of vitamins and minerals did not reflect the risk of insufficiency reported in dietary analysis. The impact of hypervitaminosis A and E is unknown and warrants exploration.

## Figures and Tables

**Table 1:** Participant characteristics.

Total	46
Age (mean; years)	10.5 (SD ± 4.2)
Gender	Male = 28 (61%), Female = 18 (39%)
eGFR (mean; mL/min/1.73 m^2^)	57.4 (SD ± 24)
CKD stage	
2	21 (46%)
3a	7 (15%)
3b	12 (26%)
4	5 (11%)
5	1 (2%)
Diagnosis	
Dysplasia	11 (24%)
Obstructive Nephropathy	8 (17%)
HUS	8 (17%)
Reflux Nephropathy	7 (15%)
Ischaemic Injury (e.g., HIE)	3 (7%)
Idiopathic	2 (4%)
Nephrotic Syndrome	1 (2%)
Syndrome-Related	1 (2%)
Tubulointerstitial Nephritis	1 (2%)
Wilms’ Tumour	1 (2%)
Kidney Venous Thrombosis	1 (2%)
Polycystic Kidney Disease	1 (2%)
Number of medications	3.9 (SD ± 2.5)
Anti-hypertensive Use	26 (57%)
Dose of enalapril (median; mg/kg)	0.18 (IQR ± 0.11)
Time since diagnosis (mean; months)	93 (SD ± 55)
Systolic blood pressure SDS	−0.52 (SD ± 0.84)
Diastolic blood pressure SDS	−0.52 (SD ± 0.50)

Demographic details of the conservatively managed subgroup only. Abbreviations: CKD—chronic kidney disease; eGFR—estimated glomerular filtration rate; IQR—interquartile range; SD—standard deviation; SDS—standard deviation score.

**Table 2:** Absolute intake and percentage of recommended intake, in comparison to recommended intake and general population intake.

Nutrient	Mean Intake ± SD	Mean Dietary Intake; % of Recommended	Difference from Recommendations	% Meeting Min Recommendations	Difference from NDNS Intake, Younger Children	Difference from NDNS Intake, Older Children
Energy (kcal/d)	1510 ±593	89.6 ± 40.9	0.09	35%	0.462	0.021 * ▼
Protein (g/d) ‡	45.7 ± 25.9	172 ± 94	<0.00001 * △	20%	0.245	0.005 * ▼
Sodium (mg/d) ‡	1699 ± 1073	170 ± 101	0.000021 * △	78%		
Potassium (mg/d) ‡	1694 ± 939	95.4 ± 65.2	0.642	26%	0.019 * ▼	0.001 * ▼
Calcium (mg/d) ‡	586 ± 519	117 ± 94	0.240	48%	0.773	0.109
Magnesium (mg/d) ‡	151 ± 85	95.1 ± 67.9	0.626	30%	0.250	0.001 * ▼
Phosphorus (mg/d) ‡	844 ± 546	173 ± 103	0.000018 * △	70%		
Vitamin A (µg/d) ‡	504 ± 436	139 ± 127	0.044 * △	52%	0.028 * △	0.597
Vitamin E (mg/d)	5.1 ± 2.4	192 ± 103	<0.00001 * △	83%		
Riboflavin (mg/d) ‡	1.29 ± 0.89	135 ± 84	0.006387 * △	65%	0.285	0.391
Folate (mg/d)	174 ± 71.6	118 ± 60	0.047816 * △	57%	0.282	0.155
Iron (mg/d) ‡	7.8 ± 4.5	97.5 ± 85.4	0.844	43%	0.080	0.196
Iodine (mg/d) ‡	82 ± 73.5	85.9 ± 65.3	0.150	26%	0.155	0.009 * ▼
Selenium (µg/d) ‡	27 ± 20.3	89.9 ± 62.2	0.264	39%	0.516	0.001 * ▼
Zinc (mg/d) ‡	5.65 ± 3.08	81.1 ± 42.3	0.004521 * ▼	30%	<0.0005 *△	0.002 * ▼
Copper (µg/d) ‡#	665 ± 390	103 ± 64	0.753	39%		
Manganese (mg/d) ‡	1.5 ± 0.8	352 ± 240	<0.00001 * △	87%		
Thiamine (mg/d) ‡	1.3 ± 0.9	219 ± 84	<0.00001 * △	93%		
Niacin (mg/d) ‡	22.4 ± 11.7	264 ± 93	<0.00001 * △	100%		
Vitamin B6 (µg/d) ‡	1.3 ± 0.9	191 ± 70	<0.00001 * △	98%		
Vitamin C (mg/d) ‡	58 ± 51.0	249 ± 239	0.000112 * △	80%		
Vitamin B12 (µg/d) ‡	2.1 ± 2.8	283 ± 254	0.000013 * △	74%		
Vitamin K (µg/d) ‡	3 ± 7	51.3 ± 195	0.097	0%		
Pantothenic acid (mg/d) ‡	3.4 ± 2.	121 ± 55	0.015121 * △	61%		
Biotin (µg/d) ‡	15.8 ± 12.4	179 ± 114	0.000025 * △	74%		
Dietary Fibre	7.5 ± 4.3	34.6 ± 20	<0.00001 * ▼	0%		

‡—Non-parametric distribution, median and interquartile range reported. * Statistically significant. △—CKD cohort with higher intake. ▼—CKD cohort with lower intake. Where RNI values exist, they were used; other requirements were estimated as follows: energy—estimated by the Henry equation [17]; manganese—16 mcg/k/d; vitamin E—ratio of polyunsaturated fatty acids of 0.4; pantothenic acid—3 µg/d; biotin—10 µg/d; and vitamin K—1 µg/k/d. #—No LRNI value available. Abbreviations: LRNI—lower reference nutrient intake; NDNS—National Diet and Nutrition Survey; RNI—reference nutrient intake. Values from the NDNS data are in Tables S2 and S3.

**Table 3:** Blood concentrations of key analytes, including vitamins and minerals.

Nutrient (Units)	Average	SD/IQR	Range Min	Range Max	Number Lower than Reference Range	Number Higher than Reference Range
Haemoglobin (g/L)	129.14	16.92	86.00	160.00	9	0
Sodium ‡ (mmol/L)	137.00	2.00	134.00	141.00	0	0
Potassium ‡ (mmol/L)	4.2	0.63	3.40	5.50	1	4
Corrected Calcium (mmol/L)	2.42	0.10	2.21	2.62	0	2
Magnesium ‡ (mmol/L)	0.82	0.13	0.71	1.21	0	2
Inorganic Phosphate (mmol/L)	1.36	0.23	0.70	1.93	1	1
Ferritin ‡ (µg/L)	45.00	84.50	14.00	488.00	0	4
Copper (µmol/L)	19.42	4.78	9.70	31.60	1	3
Zinc ‡ (µmol/L)	13.55	3.13	9.50	22.80	5	0
Manganese ‡ (nmol/L)	152.00	69.00	61.00	494.00	2	8
Selenium (µmol/L)	1.04	0.20	0.47	1.46	1	0
Vitamin A (µmol/L)	2.38	0.84	0.80	4.50	1	31
Vitamin E ‡ (µmol/L)	27.90	7.63	20.80	60.50	0	40
Vitamin B12 ‡ (ng/L)	384.00	364.25	100.00	1072.00	1	-
Folate € (ng/mL)	10.45	-	4.30	>25.00	0	6
Vitamin C ‡ (µmol/L)	70.60	42.75	6.20	200.10	1	2
Vitamin B6 ‡ (nmol/L)	80.20	39.20	19.40	253.60	1	2
Vitamin D ‡ (nmol/L)	95.00	72.00	36.00	239.00	5	-

‡—Non-parametric distribution, average expressed as median and interquartile range (IQR); otherwise, mean and standard deviation (SD) presented. As folate is expressed as >25 ng/mL for those above the reference range, SD cannot be calculated for folate. There is no upper reference range for vitamin B12 or vitamin D. €—Geometric mean reported. Vitamin A (total retinol) and vitamin E (total tocopherols) were elevated in the majority of children. There was a low prevalence of low nutrient measurements in the cohort.

## Data Availability

Data are available on request with necessary permissions.

## Supplementary Materials

The following supporting information can be downloaded at https://www.mdpi.com/article/10.3390/nu17030535/s1: Table S1: Normal reference ranges for children and young people aged 4 to 18 years; Table S2: Comparison of the TEMPeReD cohort with National Diet and Nutrition Survey data ages 4 to 10 years; Table S3: Comparison of the TEMPeReD cohort with National Diet and Nutrition Survey data ages 11 to 18 years; Table S4: Dietary intake of fibre age group. Table S5. Comparison of those with dietary intake of fibre below and above the median; Table S6. Comparison of those with a decline in eGFR at 12 months, and those without.
