# Supplementary material for: Nutritional Characterisation of Childhood Chronic Kidney Disease: Trace Element Malnutrition in Paediatric Renal Disease (TeMPeReD) Study
Source: Nutrients. 2025 Jan 31;17(3):535. doi: 10.3390/nu17030535 (PMC11820732; doi:10.3390/nu17030535)
Supplement: Supplementary file 1 [file nutrients-17-00535-s001.zip › nutrients-3392634-supplementary.pdf]

Table S1. Normal Reference Ranges for children and young people aged 4 to 18 years. .

Nutrient	Normal Reference Range
Sodium	133-146 mmol/l
Potassium	3.5 – 5.0 mmol/l
Corrected Calcium	2.2-2.6 mmol/l
Magnesium	0.7-1.0 mmol/l
Inorganic Phosphate	0.9-1.8 mmol/l
Ferritin	10-170 µg/l
Copper	12-26 µmol/l
Zinc	11-24 µmol/l
Whole Blood Manganese	73-210 nmol/l
Selenium	0.7-1.7 µmol/l
Vitamin A	0.9-1.7 µmol/l
Vitamin E	10-21 µmol/l
Vitamin B12	>130 ng/l
Folate	2.9-20.6 ng/l
Vitamin C	11-114 µmol/l
Vitamin B6	30-144 nmol/l
Vitamin D	>50 nmol/l

Table S2. Comparison of the TEMPeReD cohort with National Diet and Nutrition Survey data ages 4 to 10 years.

	**NDNS cohort**		**TEMPeReD Cohort (n=25)**				
**Nutrient**	**NDNS mean**	**NDNS SD**	**Cons cohort mean**	**Cons cohort SD**	T-value (df)	p-value	p-value (corrected)
Energy	1462	317	1527	643	T(518) = 0.736	0.462	1.000
Protein	54.1	13.2	51.2	23.6	T(518) = 1.16	0.245	1.000
Vitamin A	599	457	843.96	733.5	T(518) = 2.2	***0.028****	0.336
Riboflavin	1.45	0.53	1.31	0.85	T(518) = 1.07	0.285	1.000
Folate	183	63	173.09	72.15	T(518) = 1.08	0.282	1.000
Iron	8.2	2.6	9.61	9.59	T(518) = 1.76	0.080	0.960
Calcium	781	270	745.48	556.72	T(518) = 0.289	0.773	1.000
Magnesium	188	49	179.3	82.78	T(518) = 1.15	0.250	1.000
Potassium	2115	520	1893.22	860.46	T(518) = 2.35	***0.019****	0.228
Iodine	129	64	108.09	72.17	T(518) = 1.42	0.155	1.000
Selenium	32	11	30.35	13.58	T(518) = 0.65	0.516	1.000
Zinc	6.1	1.8	12.65	30.97	T(518) = 4.37	***0.000****	***<0.006****

Table S3. Comparison of the TEMPeReD cohort with National Diet and Nutrition Survey data ages 11 to 18 years. .

	NDNS cohort		TEMPeReD Cohort (n=21)				
**Nutrient**	**NDNS mean**	**NDNS SD**	**Cons cohort mean**	**Cons cohort SD**	T-value (df)	p-value	p-value (corrected)
Energy	1779	526	1507	577	T=(576) = 2.314	***0.021****	0.253
Protein	67.1	24.2	52.06	23.15	T=(576) = 2.799	***0.005****	0.064
Vitamin A	626	475	570.05	480.32	T=(576) = 0.5295	0.597	1.000
Riboflavin	1.48	0.73	1.34	0.81	T=(576) = 0.859	0.391	1.000
Folate	208	91	179.38	73.83	T=(576) = 1.423	0.155	1.000
Iron	9.6	3.3	8.65	3.21	T=(576) = 1.296	0.196	1.000
Calcium	706	293	600.95	317.96	T=(576) = 1.607	0.109	1.000
Magnesium	215	75	160.57	59.26	T=(576) = 3.286	***0.001****	***0.013****
Potassium	2358	770	1795.67	675.03	T=(576) = 3.298	***0.001****	***0.012****
Iodine	126	87	75.76	675.03	T=(576) = 2.628	***0.009****	0.106
Selenium	42	20	26.67	19.48	T=(576) = 3.45	***0.001****	***0.007****
Zinc	7.5	2.8	5.53	2.50	T=(576) = 3.175	***0.002****	***0.019****

Table S4. Dietary intake of fibre age group.

	Fibre intake (g/day) Mean ± SD	Fibre intake (% of requirements) Mean ± SD	Number meeting requirements.
All ages (n = 46)	7.5 ± 4.3	34.6 ± 20.0 %	0
2-5 years (n = 7)	7.2 ±3.8	47.9 ± 25.5 %	0
5-11 years (n = 18)	6.1 ±3.6	30.5 ± 17.8 %	0
11-16 years (n = 16)	9.1 ± 4.5	36.5 ± 17.9 %	0
16-18 years (n = 5)	7.4 ± 6.4	24.7 ± 21.3 %	0

Recommended dietary fibre intake: 2-5 years = 15g/day; 5-11 years = 20g/day; 11-16 years = 25 g/day; 16-18 years = 30g/day [18].

Table S5. Comparison of those with dietary intake of fibre below and above the median.

	Dietary fibre intake lower than median (n=23)	Dietary fibre intake higher than median (n=23)	Comparison (T-test / Mann-Whitney U test, p value).
**Anthropometry at baseline:**			
**Height SDS †**	-1.09 (±2.27)	-0.46 (±1.65)	0.568
**Weight SDS**	-0.43 (±2.01)	-0.44 (±1.62)	0.775
**BMI SDS**	0.40 (±1.50)	0.24 (±1.34)	0.852
**Intake (as percentage of requirements):**			
**Energy**	80.9 (±40.0)	98.4 (±40.1)	0.119
**Protein**	152 (±90.0)	191 (±96.2)	0.072
**Sodium**	130 (±109)	162 (±103)	0.297
**Potassium †**	65.0 (±48.0)	86 (±108)	0.057
**Calcium †**	78.0 (±63.0)	104 (±112)	0.282
**Magnesium †**	68.0 (±52.0)	90 (±74)	0.138
**Phosphorous †**	102 (±80.0)	130 (±120)	0.150
**Iron †**	62.0 (±56.0)	100 (±44)	***0.015****
**Copper †**	73.0 (±39.0)	111 (±46)	***0.026****
**Zinc**	73.7 (±63.3)	88.5 (±41.0)	0.166
**Manganese**	294 (±168)	410 (±304)	0.170
**Selenium**	82.8 (±64.3)	96.8 (±58.5)	0.328
**Iodine**	81.8 (±51.0)	90 (±78.0)	0.792
**Vitamin A †**	104 (±126)	109 (±75.0)	0.947
**Vitamin E: PUFA**	215 (±106)	169 (±86.1)	0.104
**Thiamine**	212 (±83.0)	226 (±86.1)	0.575
**Riboflavin**	120 (±86.2)	150 (±79.8)	0.087
**Niacin †**	265 (±103)	238 (±146)	0.860
**Vitamin B6**	185 (±82.0)	174 (±67.0)	0.267
**Vitamin B12**	247 (±234)	319 (±273)	0.391
**Folate †**	106 (±62.8)	117 (±76)	0.065
**Pantothenic Acid**	110 (±46.2)	131 (±61.9)	0.253
**Biotin †**	171 (±132)	187 (±132)	0.312
**Vitamin C †**	219 (±223)	205 (±255)	0.277
**Vitamin K †**	33.9 (±48.3)	35.0 (±147)	0.233

At 12 months follow-up:			
**Height SDS †**	-0.81 (± 2.24), n=21	-0.63 (±2.19), n=21	0.940
**Weight SDS**	-0.11 (±1.98), n=20	-0.55 (±1.66), n=21	0.549
**BMI SDS**	0.49 (±1.51), n=20	0.20 (±1.40), n=20	0.620
**Change in eGFR at 12 months**	-0.13 (±14.9), n=19	-2.31 (±12.47), n=17	0.639

**- statistical significance. † - non-parametric data, median and interquartile range given.*
Abbreviations: eGFR = estimated glomerular filtration rate, PUFA = polyunsaturated fatty acids, SDS = standard deviation scores.

Table S6. Comparison of those with a decline in eGFR at 12 months, and those without.

	Decline in eGFR at 12 months (n=19)	Absence of decline in eGFR at 12 months (n=17)	Comparison (T-test / Mann-Whitney U test, p value)
**Anthropometry at baseline:**			
**Height SDS †**	-0.63 (±1.65)	-1.13 (±2.49)	0.573
**Weight SDS**	-0.38 (±1.81)	-0.53 (±2.07)	0.778
**BMI SDS**	0.23 (±1.62)	0.39 (±1.42)	0.661
**Intake (as percentage of requirements):**			
**Energy**	99.2 (±38.7)	73.9 (±34.6)	***0.025****
**Protein †**	182 (±114)	110 (±72.0	0.071
**Sodium †**	162 (±180)	130 (±91.0)	0.081
**Potassium †**	75.0 (±114)	68.0 (±51.5)	0.594
**Calcium †**	115 (±92.0)	74.0 (±59.5)	0.156
**Magnesium †**	88.0 (±76.0)	69.0 (±50.0)	0.232
**Phosphorous †**	140 (±115)	106 (±78.5)	0.087
**Iron †**	93.0 (±47.0)	74.0 (±54.5)	0.531
**Copper †**	117 (±77.0)	76.0 (±34.5)	0.081
**Zinc**	91.0 (±43.0)	72.8 (±40.0)	0.156
**Manganese †**	283 (±278)	291 (±374)	0.975
**Selenium**	93.1 (±55.8)	86.4 (±67.6)	0.616
**Iodine †**	64.0 (±83.0)	70.0 (±54.0)	0.975
**Vitamin A †**	110 (±144)	82.0 (±104)	0.081
**Vitamin E: PUFA †**	186 (±57.0)	197(±142)	0.754
**Thiamine**	214 (±91.5)	243 (±86.6)	0.244
**Riboflavin †**	120 (±81.0)	118 (±133)	1.000
**Niacin †**	238 (±110)	288 (±125)	0.300
**Vitamin B6**	171 (±50.8)	223 (±82.3)	***0.038****
**Vitamin B12 †**	220 (±467)	160 (±199)	0.531
**Folate †**	117 (±79.0)	101 (±83.0)	0.661
**Pantothenic Acid**	130 (±55.3)	109 (±54.0)	0.271
**Biotin †**	171 (±110)	125 (±109)	0.165
**Vitamin C †**	205 (±176)	120 (±134)	***0.049****
**Vitamin K †**	13.0 (±40.0)	23.0 (±75.5)	0.950
**At 12 months follow-up:**			
**Height SDS †**	-0.63 (±1.93), n=19	-0.86 (±2.55). n=17	0.490
**Weight SDS**	-0.23 (±1.83), n=18	-0.40 (±1.98), n=17	0.660
**BMI SDS**	0.12 (±1.62), n=18	0.62 (±1.39), n=16	0.251

**- statistical significance. † - non-parametric data, median and interquartile range given.*
Abbreviations: eGFR = estimated glomerular filtration rate, PUFA = polyunsaturated fatty acids, SDS = standard deviation scores.
